# Association of Osteopontin and selective glomerular hypofiltration syndrome with hospitalization for kidney failure in acute heart failure patients

**DOI:** 10.1186/s12882-025-04481-w

**Published:** 2025-10-06

**Authors:** Marcus Andreas Ohlsson, Christopher Nilsson, John Molvin, Hannes Holm Isholth, Anders Grubb, Anders Christensson, Amra Jujic, Martin Magnusson

**Affiliations:** 1 Department of Internal Medicine, Skåne University Hospital, Ruth Lundskogs gata 3, Malmo, 205 02 Sweden; 2 Department of Clinical Sciences, Lund University, Malmö, Sweden; 3 Department of Nephrology, Skåne University Hospital, Malmö, Sweden; 4 Department of Cardiology, Lund University, Malmö, Sweden; 5 Department of Clinical Chemistry, Skåne University Hospital, Lund University, Lund, Sweden; 6 Wallenberg Centre for Molecular Medicine, Lund University, Lund, Sweden; 7 Hypertension in Africa Research Team (HART), North-West University, Potchefstroom, South Africa

**Keywords:** Kidney failure, Renal failure, Hospitalization, Heart failure, Biomarkers, Selective glomerular hypofiltration syndrome, Shrunken pore syndrome

## Abstract

**Background:**

Selective glomerular hypofiltration syndrome (SGHS), formerly shrunken pore syndrome, an emerging marker of glomerular dysfunction, is associated with worsened prognosis in cardiovascular disease, but its role in kidney failure remains underexplored. Osteopontin (OPN) is associated with worsening kidney function and prognosis in chronic kidney disease.

**Purpose:**

To explore if increased levels of OPN and SGHS are associated with hospitalization for kidney failure in patients with acute heart failure (HF).

**Methods:**

OPN was analyzed in 315 hospitalized HF patients using a proximity extension assay. SGHS was defined as cystatin C-based estimated GFR (eGFR) < 60% of the creatinine-based eGFR. Clinical outcomes were retrieved from regional registries (ICD-10 N17-N19). Multivariable logistic and Cox regression models adjusted for known risk factors were used for associations between OPN and SGHS and their association with hospitalization for kidney failure.

**Results:**

The mean age was 74 years, 33% were female and 15% presented with SGHS. Patients with SGHS had higher BMI, OPN- and cystatin C levels and higher mean eGFR. During the median follow-up period of 28 months, 46 patients were hospitalized for kidney failure. Increased OPN-levels were associated with prevalent SGHS (odds ratio 2.50, *p* < 0.001), and higher risk of hospitalization for acute kidney failure (hazard ratio (HR) 4.66, *p* < 0.001) as did prevalent SGHS (HR 4.82, *p* < 0.001).

**Conclusions:**

In HF patients, OPN was associated with a higher prevalence of SGHS and both OPN and SGHS were associated with higher risk of hospitalization for kidney failure. Our results suggest that OPN and SGHS can identify HF patients at high risk of kidney function decline.

## Introduction

Shrunken pore syndrome (SPS), first described in 2015 by Grubb et al., is characterized by a significantly lower estimated glomerular filtration rate (eGFR) based on cystatin C than on creatinine, commonly defined as eGFRcys < 70% or < 60% of eGFRcrea [1, 2]. Initially observed in pregnant women, SPS was linked to reduced elimination of larger molecules (5–40 kDa) like cystatin C, while smaller ones (< 0.2 kDa) like creatinine remained unaffected [3]. This suggests a reduction in glomerular pore diameter. SPS has been associated with increased mortality, heart failure (HF), HF rehospitalization, and end-stage kidney disease [4–10].

Current research suggests that SPS involves not only a reduction in pore size but also a change in pore morphology, resulting in elongation [3, 11]. Therefore, the concept of selective glomerular hypofiltration syndromes (SGHS) has emerged to better characterize this condition [3]. The exact mechanisms underlying this association are not fully understood but the hypofiltration that occurs in SGHS has been associated with elevated levels of several proteins that previously have been associated with atherosclerosis, a finding that may provide a clue to the connection between SGHS and cardiovascular morbidity [6, 12].

Deteriorating kidney function among patients with HF indicates advanced disease with a poor prognosis [13]. The complex mechanism behind this development is called cardiorenal syndrome (CRS), which is divided into five subtypes based on pathophysiology [14]. CRS incorporates a bidirectional dysfunction of both the heart and the kidneys [13].

In clinical practice, reliable methods for diagnosing and monitoring kidney function in HF patients are crucial. The current standard method consists of serial measurements of plasma creatinine (P-creatinine) and eGFR. Since 1995, Skåne University Hospital has used a routine eGFR assay averaging creatinine- and cystatin C-based estimates [15]. However, P-creatinine has limitations, as it rises only after a 50% GFR decline and takes 24–36 h to stabilize [16, 17]. This highlights the need for earlier detection of kidney dysfunction. Several biomarkers, including Kidney injury molecule-1 (KIM-1), Neutrophil gelatinase-associated lipocalin (NGAL), Interleukin-8 (IL-8), Human liver-type fatty acid-binding protein (hL-FABP), Tissue inhibitor of metalloproteinases-2 (TIMP-2) and proenkephalin a 119–159 (penKid), have been investigated, but none has reached clinical use [16, 18].

The proinflammatory and fibrotic protein Osteopontin (OPN) has been implicated in kidney dysfunction. While it aids in kidney repair, sustained overexpression promotes chronic inflammation and disease progression [19]. Primarily produced in the kidneys, but also in the gallbladder and brain, OPN is secreted into urine by distal nephrons and the loop of Henle, with its expression stimulated by Angiotensin II [19–21].

In this study, we aim to investigate the occurrence and impact of SGHS in patients with HF and its possible association with hospitalization for kidney failure. Moreover, we aim to investigate the associations between OPN and SGHS, as well as the possible association between OPN and hospitalization for kidney failure.

## Methods

### Study population

The HeARt and Brain Failure inVESTigation study in Malmö, Sweden (HARVEST-Malmö) is an ongoing study at the Skåne University Hospital. Patients admitted to either the Department of Internal Medicine or the Department of Cardiology for acute HF (ICD-10 I50-) are eligible for inclusion. The only exclusion criterion is the inability to give informed consent. In the case of patients with severe cognitive impairment, the consent is provided by patient’s relatives [8].

Between March 2014 and March 2024, 598 patients were included in the study. Of these, 570 cases had complete data for analyses of associations between SGHS and kidney failure hospitalization. Three-hundred-twenty-four cases had available proteomic data at baseline examination. Of the 324 available cases with proteomic data, 315 cases also had complete data for the endpoint of subsequent hospitalization for kidney failure and were examined further in this study. The study was approved by the Ethical review board at Lund University, Sweden. Written informed consent was obtained from all participants.

### Clinical examination

Participants were examined with anthropometric measurements upon admission to the ward. Blood pressure was measured using a validated automated blood pressure monitor (Boso Medicus, Bosch and Sohn, Jungingen, Germany) after a 10-minute rest.

### Definitions

The diagnosis of heart failure was based on a combination of diagnostic imaging, NTproBNP-levels and/or the clinical assessment of the attending physician resulting in the diagnostic ICD-10 codes of I50.0-I50.9. Prevalent SGHS was defined as eGFR_cys_/eGFR_crea_ <0.6 [9]. Body mass index (BMI) was defined as kg/m^2^. “Acute kidney failure” included ICD-10 codes N17.0-N17.9; N17.0: Acute kidney failure with tubular necrosis, N17.1: Acute kidney failure with acute cortical necrosis, N17.2: Acute kidney failure with medullary necrosis, N17.8: Other acute kidney failure and N17.9: Acute kidney failure, unspecified. “All kidney failures” was defined as ICD-10 codes N17.0-N17.9 as mentioned above and including chronic kidney disease N18-18.5, N18.9: Chronic kidney disease, unspecified and N19: Unspecified kidney failure.

Use of Renin-angiotensin-aldosterone system inhibitors (RAAS-inhibitors) was defined as use of either angiotensin converting enzyme inhibitor or angiotensin receptor blocker. Use of diuretics was defined as the use of either loop- or thiazide diuretics.

### Laboratory assays

Blood samples were collected at admission as well as during hospital stay in a fasting condition. Blood samples were stored at − 80 °C. Plasma cystatin C was analyzed at the Department of Clinical Chemistry, Skåne University Hospital, using an automated particle-based immunoassay, adjusted to the international reference preparation ERM-DA 471/IFCC, (Hitachi Modular P analysis system; Roche, Basel, Switzerland). P-creatinine was measured using an enzymatic colorimetric assay with an IDMS-traceable calibrator on the Hitachi Modular P analysis system (Roche, Basel, Switzerland). The revised Lund-Malmö equation was used to calculate eGFR_crea_ [22] and the Caucasian-Asian-Pediatric-Adult (CAPA) equation was used to calculate eGFR_cys_ [23]. A proximity extension array technique, using Proseek Multiplex CVD III 96 × 96 reagents kit (Olink, Uppsala, Sweden) was used for determination of plasma concentrations of OPN. The final proteomic assay readout was given as normalized protein expression, an arbitrary unit given on a log2 scale meaning that each unit increase corresponds to a doubling in concentration.

### Statistics

All statistical analyses were conducted using R Statistical Software (version 4.4.1; R Core Team, 2021). Between-group comparisons were performed utilizing Welch’s bootstrap method, with 9,999 resampling iterations. Chi-square (χ²) or Fischers exact tests were applied to evaluate differences in binary variables and Student’s T-test or Mann-Whitney U-test were applied to evaluate differences in continuous variables, where appropriate.

For handling missing values in specific variables, systolic blood pressure (*n* = 8) and eGFR (*n* = 8), the ‘mice’ package (version 3.16.0) was used; imputation was not applied to other variables. Descriptive analyses, such as counts of missing values and survival counts, were conducted using base R functions alongside the ‘dplyr’ package (version 1.1.1). Predictive Mean Matching was employed as the imputation technique to produce five multiple imputation datasets, which were then pooled to generate a complete dataset for further analysis. Kaplan-Meier survival curves were generated for the SGHS and non-SGHS groups using the ‘survival’ package (version 3.5.0) and visualized with ‘survminer’ (version 0.4.9). The cumulative probabilities of hospitalization and median survival times were calculated using the ‘ggsurvplot()’ function.

To examine the effect of SGHS on the risk of hospitalization due to kidney failure, Cox proportional hazards regression models were used and to examine the effects of OPN on the presence or absence of SGHS at baseline, logistic regression analysis was used. All multivariate models were adjusted for age, sex, BMI, systolic blood pressure, and kidney function (eGFR). Model results were summarized using the ‘gtsummary’ package (version 1.6.0). Data visualizations, including Kaplan-Meier and density plots for eGFR distributions between SGHS and non-SGHS groups, were created using the ‘ggplot2’ package (version 3.4.0). The pooled outcomes of the Cox regression models were organized into tables using the ‘flextable’ package (version 0.6.7), with formatting and exportation handled by the ‘officer’ package (version 0.6.7). No violations of proportional hazards assumptions were identified. A significance level of *p* < 0.05 was applied throughout the analyses.

## Results

Basic characteristics of the cohort are presented in Table 1. Of the total number of 570 patients, 83 patients fulfilled the criteria of SGHS upon inclusion and 487 were free from SGHS. With regards to the total cohort, the mean age was 74 years and two thirds were male.

**Table 1:** Characteristics of the study population at baseline

	Total	SGHS	Free from SGHS	*p*
	*n* = 570	*n* = 83	*n* = 487	
Age (years)	74.3 (± 12.2)	73.1 (± 10.0)	74.5 (± 12.6)	0.341
Male sex	384 (67.4)	59 (71.1)	325 (66.7)	0.435
BMI (kg/m^2^)	28.0 (± 6.1)	29.7 (± 7.6)	27.7 (± 5.8)	0.006
SBP (mmHg)	137.2 (± 27.8)	131.9 (± 26.1)	138.2 (± 28.0)	0.057
RAAS inhibitors (*n*; %)	440 (77.2)	63 (75.9)	377 (77.4)	0.480
Diuretics (*n*; %)	546 (95.8)	81 (97.6)	465 (95.5)	0.161
MRA (*n*;%)	79 (13.9)	11 (13.3)	68 (14.0)	0.878
NYHA-class III-IV (*n*; %)	443 (77.7)	59 (71.1)	384 (78.9)	0.177
Ischemic etiology of heart failure (*n*; %)	185 (32.5)	21 (25.3)	164 (33.7)	0.511
LVEF (%) (*n* = 335)	38 (± 15)	43 (± 15)	38 (± 16)	0.054
NT-proBNP (ng/L)	4453 (2212–8981)	5111 (2012–8911)	4370 (2227–9055)	0.706
Cystatin C (mg/L)	1.67 (1.34–2.18)	2.12 (1.79–2.45)	1.59 (1.29–2.08)	< 0.001
Creatinine (µmol/L)	106 (84–139)	101 (77–128)	106 (85–140)	0.127
eGFR*	51 (36–65)	54 (45–68)	50 (35–64)	0.008
Hospitalization for all kidney failures (n; %)	46 (8.1)	7 (8.4)	39 (8.0)	0.895
Hospitalization for acute kidney failure (n; %)	27 (4.7)	7 (8.4)	19 (3.9)	0.067
OPN-concentration (AU) (*n* = 315)	6.88 (± 0.76)	7.13 (± 0.69)	6.84 (± 0.76)	0.034

* eGFR is the mean of eGFR based upon cystatin C or upon creatinine using the CAPA [23] and Lund/Malmö revised formula [22], respectively; This eGFR value is the closest to measured GFR, including in SPS and SGHS. SGHS – selective glomerular hypofiltration syndrome; BMI – body mass index; SBP – systolic blood pressure; RAAS – Renin-Angiotensin-Aldosterone-system; NYHA-class – New York Heart Association Classification; NT-proBNP - N-terminal pro B-type natriuretic peptide; LVEF - left ventricle ejection fraction; eGFR – estimated glomerular filtration rate

**Fig. 1:**
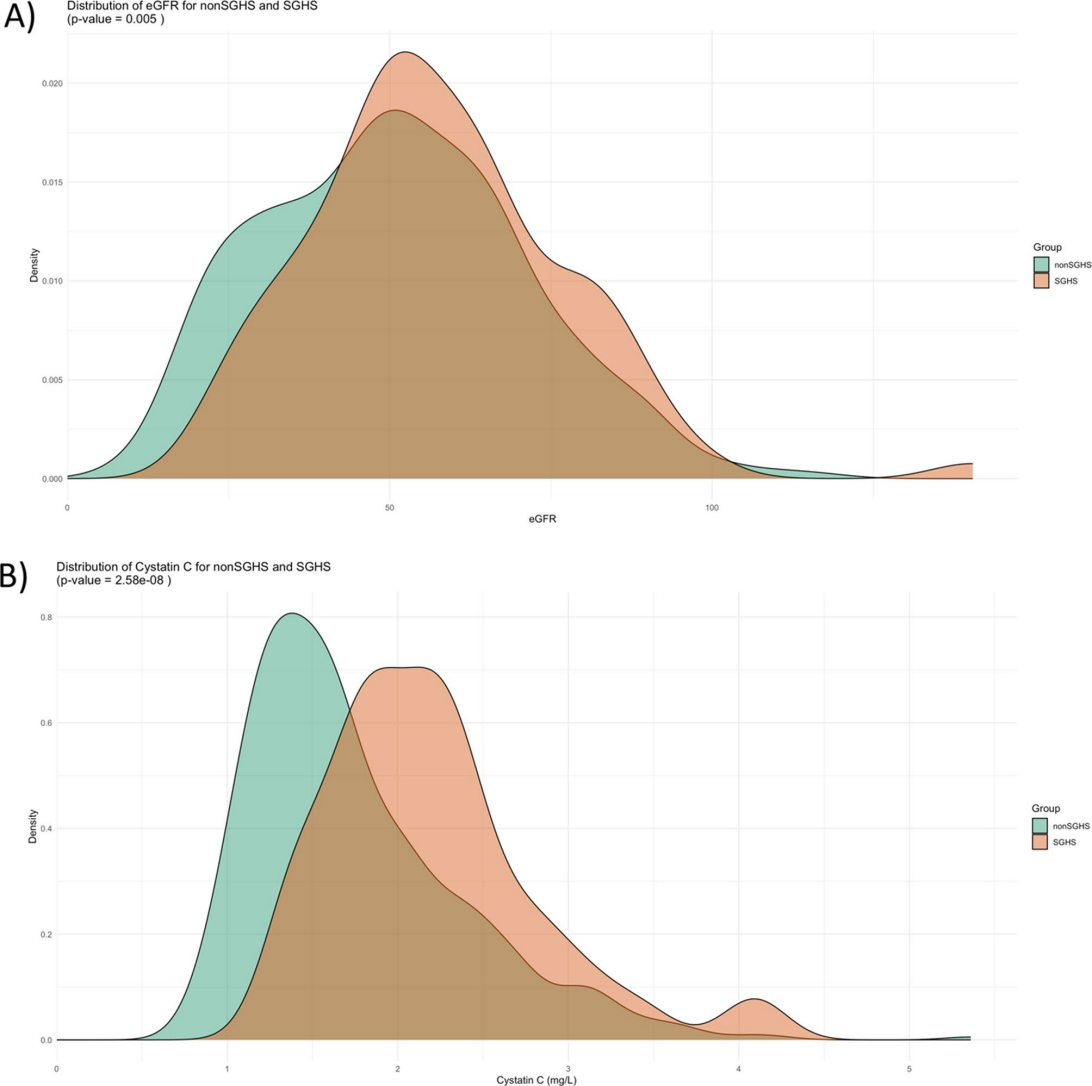

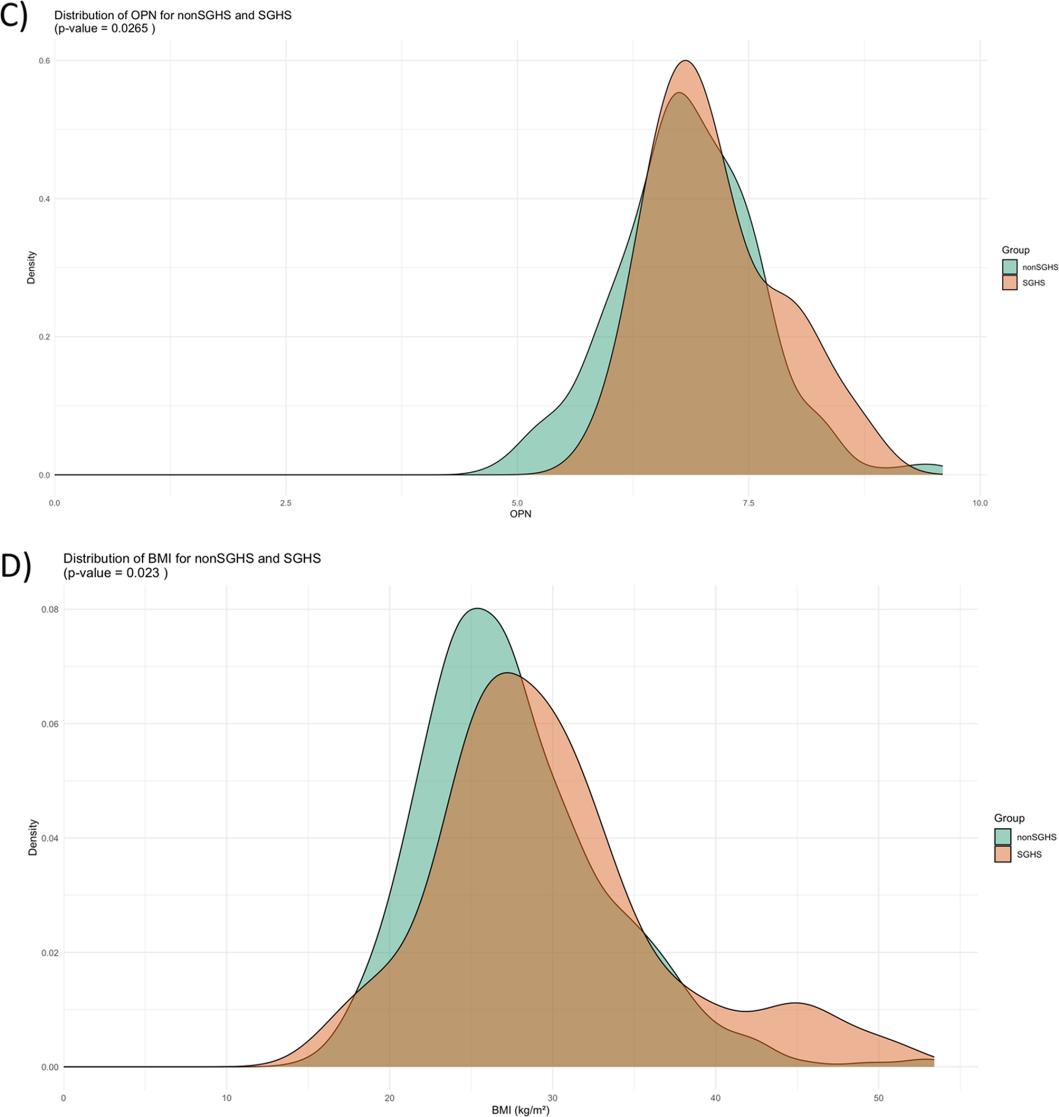
**A-D.** Illustrations showing distribution of estimated glomerular filtration rate (eGFR), cystatin C, Osteopontin and Body Mass Index (BMI) in subjects with and without selective glomerular hypofiltration syndrome (SGHS)

During the follow-up time (median 27.9 months, 25–75 interquartile range (IQR) 10.0–61.6), 46 participants were hospitalized for all kidney failures (ICD-10 codes N17-N19). For acute kidney failure (ICD-10 code N17), the follow-up time had a median of 28.9 months (IQR 10.1–63.7), and 27 patients experienced hospitalization with ICD-10 code N17 as the main diagnosis. Patients presenting with SGHS upon inclusion had significantly higher BMI (kg/m^2^) and eGFR (mL/min/1.73m^2^) as well as higher cystatin C (mg/L), and OPN-concentrations (Fig. 1A-D), otherwise no significant differences were observed between the groups.

Exploring ischemic vs. non-ischemic heart failure, no significant differences were seen concerning hospitalization for acute kidney failure (*p* = 0.909) or hospitalization for all kidney failures (*p* = 0.581). Furthermore, no significant differences were seen in OPN-concentrations between ischemic vs. non-ischemic etiology (*p* = 0.774). Multivariable Cox regression analysis showed that patients presenting with SGHS upon inclusion were more likely to be hospitalized for kidney failure (Table 2).

**Table 2:** Associations between selective glomerular Hypofiltration syndrome and hospitalization for acute or all kidney failures

	All kidney failures			Acute kidney failure		
	HR	95%CI	*p*	HR	95%CI	*p*
SGHS	2.80	(1.18, 6.61)	0.019	4.82	(1.91, 12.19)	< 0.001
Age	0.97	(0.94, 1.00)	0.021	0.99	(0.95, 1.03)	0.463
Sex	1.24	(0.64, 2.37)	0.525	1.77	(0.77, 4.07)	0.177
SBP	1.00	(0.99, 1.01)	0.558	1.00	(0.99, 1.01)	0.767
eGFR	0.93	(0.91, 0.95)	< 0.001	0.96	(0.93, 0.98)	< 0.001
BMI	1.03	(0.98, 1.08)	0.293	1.03	(0.98, 1.10)	0.263

Values are hazard ratios (HR) and 95% confidence intervals for hospitalization for all kidney failures (ICD-10 codes N17-N19), or acute kidney failure (ICD-10 code N17). SBP – systolic blood pressure, eGFR - based on CAPA Lund/Malmö Revised formula combining creatinine and cystatin C, BMI – body mass index

**Fig. 2:**
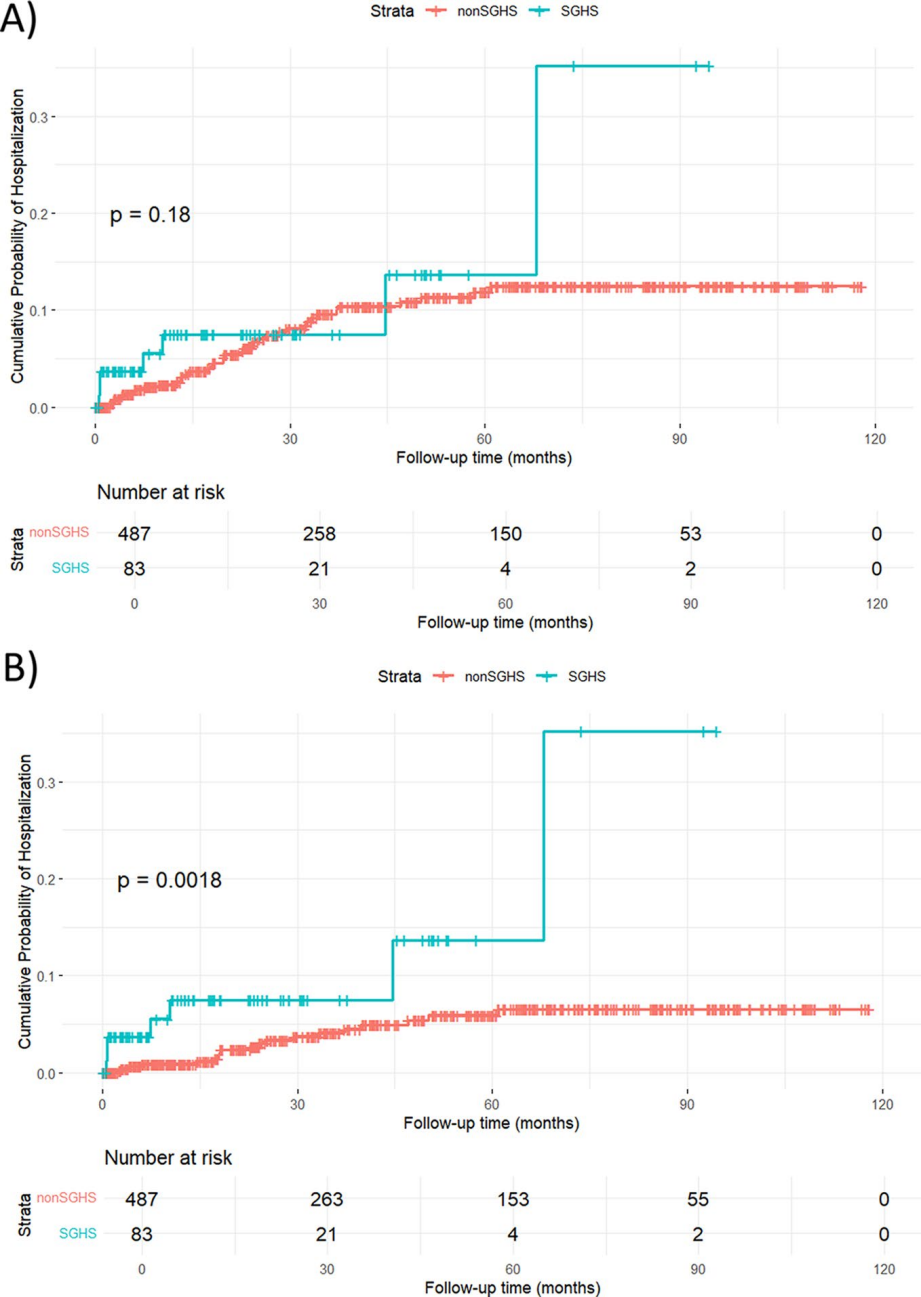
(**A**) Cumulative probability of hospitalization for all kidney failures in subjects with and without SGHS. (**B**) Cumulative probability of hospitalization for acute kidney failure in subjects with and without SGHS

The cumulative probability for hospitalization for kidney failure for SGHS versus non SGHS is illustrated in Fig. 2A and B.

Furthermore, higher OPN-levels upon baseline were significantly associated with the presence of SGHS at baseline (Table 3).

**Table 3:** Associations between OPN and SGHS at baseline

	OR	95%CI	*p*
OPN	2.50	1.47, 4.27	< 0.001
Age	1.01	0.97, 1.05	0.698
Sex	0.63	0.26, 1.55	0.318
SBP	1.00	0.99, 1.01	0.853
eGFR	1.04	1.01, 1.06	0.002
BMI	1.05	0.99, 1.12	0.104

Values are odds ratios (OR) and 95% confidence intervals for hospitalization for all kidney failures (ICD-10 codes N17-N19), or acute kidney failure (ICD-10 code N17). SBP – systolic blood pressure, eGFR - based on CAPA Lund/Malmö Revised formula combining creatinine and cystatin C, BMI - body mass index

Multivariable Cox regression analysis showed that OPN was associated with hospitalization for all kidney failures (26 events, 280 controls, Table 4) as well as hospitalization for acute kidney failure (16 events, 290 controls).

**Table 4:** Associations between OPN and hospitalization for all kidney failures or acute kidney failure

	All kidney failures			Acute kidney failure		
	HR	95%CI	*p*	HR	95%CI	*p*
OPN	3.31	(1.72, 6.38)	< 0.001	4.66	(2.11, 10.30)	< 0.001
Age	0.96	(0.93, 0.99)	0.008	0.97	(0.93, 1.00)	0.077
Sex	1.59	(0.67, 3.75)	0.291	1.85	(0.63, 5.46)	0.266
SBP	0.99	(0.98, 1.01)	0.412	1.00	(0.99, 1.02)	0.930
eGFR	0.97	(0.94, 0.99)	0.016	0.99	(0.96, 1.02)	0.578
BMI	1.05	(0.98, 1.13)	0.148	1.06	(0.98, 1.15)	0.139

Values are hazard ratios (HR) and 95% confidence intervals for hospitalization for all kidney failures (ICD-10 codes N17-N19), or acute kidney failure (ICD-10 code N17). SBP – systolic blood pressure, eGFR - based on CAPA Lund/Malmö Revised formula combining creatinine and cystatin C, BMI – body mass index

## Discussion

To our knowledge, this is the first study to demonstrate an association between SGHS and kidney failure hospitalization in patients with HF. Furthermore, we demonstrate that increased levels of OPN at baseline, are not only associated with the presence of SGHS, but also with higher risk of future hospitalization for kidney failure.

SGHS has previously been shown to be associated with severe eGFR decline and increased incidence of end-stage kidney disease in patients with IgA nephropathy and membranous nephropathy [24]. In a study by Zhou et al., patients with both SGHS and CKD exhibited the highest risk of long-term mortality. In the same study, it was also demonstrated that kidney transplant patients with SGHS had a higher incidence of delayed graft function and showed worse long-term kidney function, suggesting that SGHS is associated with poor post-transplant outcomes [25].

Deteriorating kidney function is a well-known and dreaded complication to HF, as a part of the cardiorenal syndrome. Exploring new ways of early detection of kidney function deterioration is essential due to limitations of traditional ways of kidney function monitoring by means of serial measurements of creatinine. In SGHS and HF, it’s possible that extrarenal factors such as sarcopenia lead to falsely high eGFR_crea,_ and subsequently SGHS. However, previous studies have shown that the association also prevails when adjusting for those factors [7]. Although we did not have data on sarcopenia status in our study, the SGHS group exhibited significantly higher BMI which is linked to lower rates of sarcopenia [26]. Furthermore, higher levels of cystatin C itself adjusting for extrarenal factors, have been recognized as a predictor of worse overall outcomes in several different populations including subjects with HF [27, 28]. Patients with acute HF are often subject to intensive decongestive treatment with diuretics, which in turn may cause kidney dysfunction. Although theoretically plausible, no such difference in use of diuretics was seen in our study between those presenting with SGHS and those who did not. Neither did we see a difference concerning the use of RAAS-inhibitors or MRA.

The imbalance in glomerular filtration posed by SGHS may provide early insight into disruptions in the glomerular filtration as a potential pathophysiological mechanism, which can subsequently impact overall kidney health and clinical outcomes. SGHS is defined as reduced filtration of molecules between 5 and 30 kDa and of the total human proteome, around 36% of its proteins have a mass below 30 kDa. Hence, reduced filtration plays an immense role in regulating the proteome. For instance, a previous study on HF subjects in the HARVEST cohort, revealed increased levels of proteins linked to arteriosclerosis among subjects with SGHS [12]. Research on proteomics and CKD has revealed several proteins related to higher risk of CKD progression. Of interest, this includes proteins that are shown to play an intrinsic role in modulating extracellular matrix [29]. Although further studies are needed, this may present a possible mechanism in SGHS rendering accumulation of proteins that may trigger structural change and fibrosis of the kidneys and ultimately a reduction in the overall GFR. The compromised filtration efficiency may therefore accelerate the progression of CKD as well as augmenting the risk of acute kidney injury (AKI). One histological study on SGHS has so far been conducted establishing an association between SGHS and structural changes in the kidneys, revealing thickened glomerular basement membrane among SGHS subjects with diabetes [11]. The potential role of SGHS as an underlying pathophysiological mechanism should be further investigated.

Osteopontin is a multifunctional glycoprotein that is involved in various biological processes including inflammation, fibrosis, and immune modulation, which are all relevant to kidney pathology. OPN is known to be a potent pro-inflammatory cytokine that recruits and activates macrophages and other immune cells. Osteopontin-driven inflammation might contribute to such glomerular injury by promoting macrophage infiltration, which can lead to glomerular scarring or fibrosis, ultimately affecting the size-selective barrier of the glomerular filtration system. This has especially been studied in diabetic kidney disease (DKD) revealing high OPN expression in the glomeruli and tubular epithelium in mouse models. Further, deletion of OPN in this study led to decreased albuminuria, mesangial area and glomerular collagen IV also highlighting OPN as a potential therapeutic target [30]. As mentioned previously, a kidney biopsy study has shown a strong correlation between the eGFR_cys_/eGFR_crea_-ratio and thickening of the glomerular basement membrane, suggesting a direct pathophysiological role in in increasing the diffusion length [11]. Hence, OPN might be directly involved in the development of SGHS by its direct effects on the glomeruli.

The link between OPN and HF has been thoroughly investigated in recent years. Studies indicate that OPN is expressed in several different cell types in the heart; cardiomyocytes, macrophages, fibroblasts and cardiac endothelial cells. The function of OPN has been linked to different cardiac regulatory processes such as fibrogenesis, vascularization, inflammation and hypertrophy and subsequently into different entities of cardiac disease such as dilated cardiomyopathy, ischemic heart disease, atrial fibrillation, valvular heart disease and right ventricular failure, although specific molecular mechanisms and their potential translation into clinical practice is still undetermined [31]. Specifically, the mechanism of OPN’s effects on acute heart failure, as observed in the current study, remains unknown.

In the current study, SGHS, or hypofiltration syndrome, defined as eGFR_cys_/eGFR_crea_<0.6, emerges as a new way of detecting kidney dysfunction and subsequent kidney failure hospitalizations among patients with HF. Presence of SGHS at baseline is in multivariate analysis strongly associated with future kidney failure hospitalization independently of eGFR and other risk factors such as sex, age, BMI and systolic blood pressure. We also propose that OPN may play a pathophysiological role in the development of SGHS and hence mediate the increased risk of AKI. Considering the possible relationship between OPN and HF, OPN may play a role in the cardiorenal syndrome and the bidirectional dysfunction.

### Strengths and limitations

One strength of this study is its relatively large cohort size, which enhances the reliability of the findings. Additionally, the cohort design closely mimics real-world clinical settings, which supports the generalizability of the results to everyday clinical practice. The study also utilizes hard endpoints, such as long-term kidney outcomes, which strengthens the robustness of the conclusions.

One limitation of our study is the presence of many unspecific kidney failure diagnoses, which may have affected our ability to define a clean and homogeneous phenotype for analysis. However, we attempted to address the issue of unspecific kidney failure diagnoses by conducting separate analyses focusing exclusively on acute kidney failure, with unchanged results. Although including unspecified and chronic kidney failures in the analyses might pose a limitation, we believe that excluding those subjects would misrepresent the broader patient population and reduce the real-world applicability of our findings. Furthermore, the findings may not be applicable to all populations, as the cohort may not adequately represent certain demographic or regional variations.

Although the group comparison analysis of hospitalization for acute kidney failure did not yield significant results (*p* = 0.067) between those with SGHS and those without SGHS we decided to move on with a multivariate analysis. This, since many of the patients included have been so recently, thus the endpoint of hospitalization has yet to occur. Previous analyses with fewer individuals (abstract presented at the European Society of Cardiology 2024) showed a clear difference between the groups, in which the SGHS-group were more prone to subsequent hospitalization. We chose to include as many patients as possible into the current study, thus lacking follow-up data on those subjects recently included. Another limitation is the lack of information concerning the etiology of kidney failure, as no information is available other than the ICD-10-codes extracted from regional registries. Finally, another limitation of the study is the lack of a replication cohort.

## Conclusion

In the current study we demonstrate for the first time, that OPN is associated with SGHS and an increased risk of future kidney failure hospitalization among patients with heart failure. Moreover, we show that the presence of SGHS at baseline, is also associated with future kidney failure hospitalization. These findings have the potential to identify heart failure patients at risk of complications requiring hospitalization due to kidney damage but warrant further investigation in order to be utilized in this aspect.

## Data Availability

The data that support the findings of this study are not publicly available due to privacy reasons but are available from the corresponding author upon reasonable request.

## Abbreviations

SGHS: Selective glomerular hypofiltration syndrome
OPN: Osteopontin
HF: Heart failure
eGFR: Estimated glomerular filtration rate
OR: Odds ratio
HR: Hazard ratio
SPS: Shrunken pore syndrome
CVD: Cardiovascular diseases
CRS: Cardiorenal syndrome
P-creatinine: Plasma creatinine
KIM-1: Kidney injury molecule-1
NGAL: Neutrophil gelatinase-associated lipocalin
IL-8: Interleukin-8
hL-FABP: Human liver-type fatty acid-binding protein
TIMP-2: Tissue inhibitor of metalloproteinases-2
penKid: Proenkephalin a 119–159
HARVEST-Malmö: The HeARt and Brain Failure inVESTigation study in Malmö, Sweden
BMI: Body mass index
RAAS-inhibitors: Renin-angiotensin-aldosterone system inhibitors
CAPA: Caucasian-Asian-Pediatric-Adult
SBP: Systolic blood pressure
NYHA-class: New York Heart Association Classification
MRA: Mineralocorticoid receptor antagonist
